# Metallic aluminium in municipal solid waste incineration fly ash as a blowing agent for porous alkali-activated granules

**DOI:** 10.1098/rsos.240598

**Published:** 2024-08-21

**Authors:** Tero Luukkonen, Yangmei Yu, Suman Kumar Adhikary, Sami Kauppinen, Mikko Finnilä, Priyadharshini Perumal

**Affiliations:** ^1^ Fibre and Particle Engineering Research Unit, University of Oulu, Oulu, Finland; ^2^ Research Unit of Health Sciences and Technology, University of Oulu, Oulu, Finland

**Keywords:** alkali-activated materials, adsorbents, artificial aggregates, fly ash, granulation, geopolymers

## Abstract

Porous alkali-activated materials are synthetic aluminosilicates that should be often produced as granules for practical applications. In the present study, municipal solid waste incineration fly ash with ~1.2 wt% of metallic aluminium was used as a novel blowing agent for metakaolin (their ratio ranged from 0% to 100%) with an aqueous sodium silicate solution as the alkali-activator and granulation fluid in high-shear granulation. The compressive strength of all granules was sufficient (≥2 MPa). Water absorption indicated an increase in porosity as the fly ash content increased. However, X-ray microtomography imaging showed no clear correlation between the fly ash content and porosity. The granules exceeded the leaching limits for earth construction materials for antimony, vanadium, chloride and sulphate. Of those, antimony, chloride and sulphate could be controlled by decreasing the ash content, but the source of vanadium was identified as metakaolin. The increase in the fly ash content decreased the cation exchange capacity of the granules. In conclusion, the recommended fly ash content is equivalent to 0.3 wt% of Al^0^ and the developed granules could be best suited as light-weight artificial aggregates in concrete where the additional binder would provide stabilization to decrease the leaching.

## 1. Introduction

Alkali-activated materials (AAMs) and geopolymers are recognized as an alternative low-CO_2_ binder for Portland cement in concrete [1]. The environmental benefits of AAMs arise from the possibility to use waste materials for their manufacturing or if natural minerals (such as 1:1 clays) are used, they require much lower calcination temperature (frequently 500−800°C) compared to Portland cement clinker preparation (1400−1500°C) [2,3]. In addition to concrete binder, AAMs have many other applications as well, for example, light-weight artificial aggregates [4], catalyst supports [5,6], adsorbents for wastewater treatment [7], pH regulating materials [8], carrier media for fixed-film bioreactors [9] or slow-release fertilizers [10]. A common feature of the aforementioned applications is that they require highly porous AAMs, often preferably in a granular form.

The typical preparation process for AAMs is to mix an aluminosilicate precursor, an alkali-activator solution, possible aggregates and admixtures; cast the material into a mould; and allow it to harden at approximately 20−80°C temperature. The methods to prepare highly porous AAM granules (or microspheres as they are commonly referred to) are, however, more complicated. One commonly used preparation approach is called the suspension–solidification method, in which the precursor, alkali-activator and surfactant are mixed vigorously to introduce air bubbles or, alternatively, a blowing agent (e.g. H_2_O_2_) is used, and then the paste is injected dropwise into a polyethylene glycol medium heated under a water bath or silicone oil [11–17]. The metakaolin-based granules formed by the suspension–solidification with 2−4 mm diameter had a mesoporous structure, specific surface area of ~54 m^2^ g^−1^, porosity of ~60% and promising adsorption capacity for Cu(II) (~35 mg g^−1^), Pb(II) (~45 mg g^−1^) and Ca(II) (~24 mg g^−1^) [11]. Another option to prepare porous granular AAMs is to use the direct foaming method, in which a blowing agent (such as H_2_O_2_) is added to the fresh-state AAM paste to generate pores, the AAM is allowed to harden and then crushed into the wanted particle size [18]. Metakaolin-based geopolymer foam crushed to 3−8 mm diameter had a mesoporous structure, specific surface area of ~39 m^2^ g^−1^, porosity of up to 71% and ammonium adsorption capacity of ~47 mg g^−1^ [18]. Alternatively, the fresh-state paste can be cast into small granule-shaped moulds [19,20] or injected on a hydrophobic surface before curing [21]. With the direct moulding method, metakaolin-based geopolymer resulted in a macroporous structure (average pore diameter ~56 nm), porosity of 63%, specific surface area of 55 m^2^ g^−1^ and Cu(II) adsorption capacity of ~21 mg g^−1^ [19]. When the granules were produced with the injection on a hydrophobic surface, the granules had a diameter between 3 and 6 mm, microporous structure and porosity of up to 75% [21]. However, these methods are cumbersome to scale-up in industrial production. Recently, the authors introduced a new method called a combined granulation–alkali activation–direct foaming process in which the precursor and powdered alkali activator are placed into a granulator and H_2_O_2_ solution is added dropwise [22]. In this process, water in the H_2_O_2_ solution causes particle wetting, dissolution of reactive aluminosilicate from the surfaces of particles by the action of the alkali activator, decomposition of H_2_O_2_ into O_2_ gas bubbles at high pH, and, finally, the aluminosilicate gel formation [4]. With the combined granulation–alkali activation–direct foaming process, metakaolin-based granules with 2−4 mm diameter had specific surface area of up to ~26 m^2^ g^−1^, microporous structure, 72% porosity and ammonium adsorption capacity of ~47 mg g^−1^ as a powder in a batch equilibrium system or ~15 mg g^−1^ as granules in a dynamic flow-through system [22].

As can be seen from the above summary, the properties of the granules are not markedly different from each other regardless of the preparation method. Thus, the feasibility of the AAM granule development for practical use is largely governed by the selection of production method (e.g. easy up-scalability) and chemicals (e.g. blowing agent). In the present study, a new process to prepare porous alkali-activated granules is studied: an aluminosilicate precursor and a solid-state blowing agent are introduced into a granulator and alkali-activator solution is added dropwise as the granulator is running. There are no previous studies in which a solid blowing agent has been used in the combined granulation and alkali-activation process. Another novel aspect of the present study is that a waste-based material, municipal solid waste incineration fly ash (MSWFA), containing metallic aluminium (Al^0^), was used as a blowing agent and co-precursor for metakaolin-based AAMs in the granulation process. Al^0^-based blowing agents are known to form Al(OH)_3_ which decreases the dissolution rate of the precursor and delays the strength development but protects the formed gel from carbonation and improves the gel connectivity [23]. The motivation of the present study was to examine whether synthetic Al^0^ could be replaced by the abundant waste material, MSWFA. More than 250 Mt of municipal solid waste is generated per year in the EU of which approximately 25 wt% is currently incinerated [24]. The proportion of incinerated municipal solid waste is expected to increase since landfilling (currently approx. 23 wt%) is strongly discouraged [24]. It is estimated that approximately 2.25 Mt of MSWFA is generated annually in the EU area [25] containing a significant amount of Al^0^ (i.e. approx. 0.027 Mt if assuming the same Al^0^ content as in the ash of the present study). Thus, new utilization prospects for MSWFA are needed.

The objectives of this study were to (i) demonstrate the pore formation of granules with MSWFA, (ii) characterize the mechanical, chemical and physical properties of the porous granules, and (iii) study the stability of granules in terms of leaching of potentially toxic elements. It should be noted that municipal solid waste incineration ashes have been studied as a pore-forming agent in the AAMs prepared via a conventional casting procedure [26] but not in the context of granulation. Thus, the present study provides useful new insights about the preparation of porous AAM granules employing solid blowing agents.

## 2. Material and methods

### 2.1. Materials and chemicals

**Figure 1.**
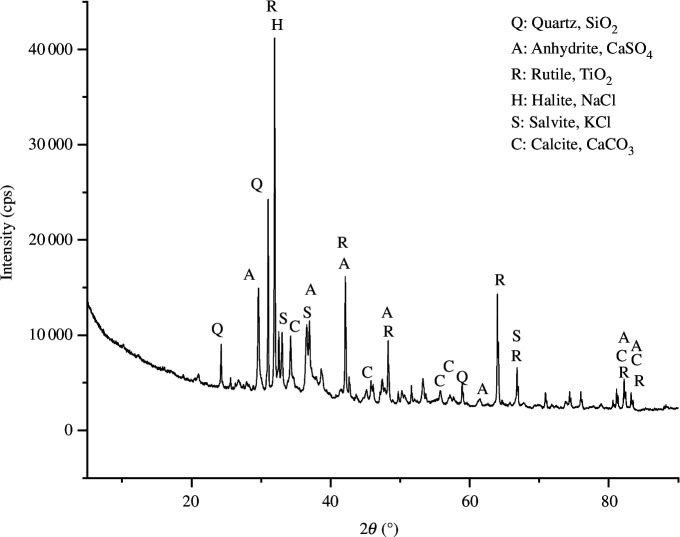
X-ray diffractogram of MSWFA.

The ash used as a blowing agent (and partial aluminosilicate precursor) was MSWFA obtained from the Laanila power plant in Oulu, Finland, which processes non-recyclable waste into steam and heat. The main aluminosilicate precursor was metakaolin (MetaMax, BASF, Germany). The composition of MSWFA and metakaolin are shown in table 1 as detected with X-ray fluorescence spectrometer (PanAnalytical Minipal 4). The X-ray diffractogram of MSWFA (detected with Rigaku SmartLab 9 kW) is shown in figure 1: the crystalline phases of MSWFA consisted of quartz (SiO_2_), rutile (TiO_2_), soluble salts (NaCl and KCl) and carbonation products (CaCO_3_). The alkali-activator solution was prepared by mixing sodium silicate solution (7.5−8.5 wt% of Na_2_O and 25.5−28.5 wt% of SiO_2_, Merck, Germany) and sodium hydroxide pellets (≥98 wt%, VWR Chemicals, Sweden) in a weight ratio of 1.00 to 0.15, respectively, for 24 h before use.

**Table 1.** Composition (the main elements as oxides, detected with XRF) of MSWFA and metakaolin employed in the preparation of granules (LOI = loss on ignition). The data of metakaolin are from [27].

composition [wt%]	MSWFA	metakaolin
Al_2_O_3_	9.10	43.8
CaO	26.70	0.02
SiO_2_	26.60	53.0
MgO	3.10	0.03
Fe_2_O_3_	2.90	0.43
SO_3_	7.50	0.03
LOI at 525°C	0.80	—
LOI at 950°C	6.20	0.46
Al^0^	1.25	—

### 2.2. Preparation of porous granules

Granules were prepared using a high shear granulator (Eirich EL1, Germany) with a tilting angle of 45° angle, rotation speed of 1200 rpm and granulation time of 20 min per batch. The weight proportions of metakaolin and MSWFA were varied according to table 2 (their total mass was 200 g per batch). The alkali-activator solution was dosed dropwise to the mixture of metakaolin and MSWFA in the granulator until the granules were formed as determined by a visual observation. The alkali-activator solution amounts shown in table 2 reflect the maximum amount that could be added before the formed granules became too wet and agglomerated together. It should be noted that the required amount of the granulation fluid is highly material specific. Granules were cured at 60°C for 4 h in a closed bag to prevent water evaporation and sieved into the particle size between 1 and 4 mm.

**Table 2.** The mixing proportions of metakaolin and MSWFA and the required amount of alkali-activator solution per each batch.

sample ID	metakaolin [wt%]	MSWFA [wt%]	alkali-activator solution [g]	Al^0^ [wt% of solid precursors]
MK100	100	0	133	0.0
MK80	80	20	133	0.3
MK60	60	40	132	0.5
MK40	40	60	95	0.8
MK20	20	80	81	1.0
MK0	0	100	48	1.3

### 2.3. Characterization methods

#### 2.3.1. Metallic aluminium

**Figure 2.**
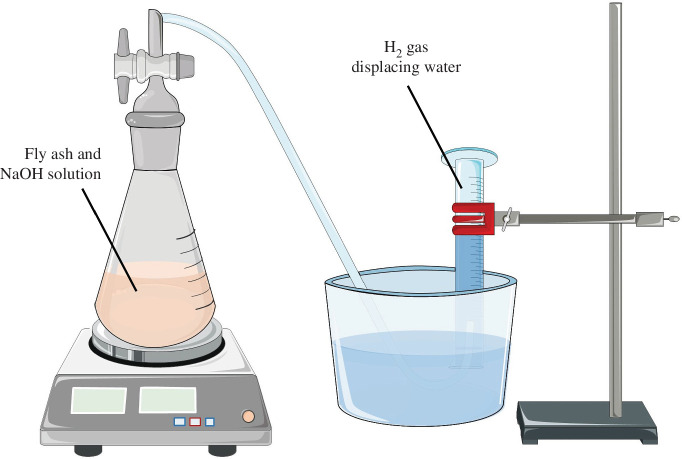
Schematic presentation of the set-up used for the Al^0^ quantification.


Al^0^ was detected by using the water displacement method in which Al^0^ is oxidized in an alkaline environment, resulting in the release of H_2_ gas (equation (2.1)). The volume of water displacement was measured and the displacement volume was used to calculate the Al^0^ content. In this experiment, the set-up shown in figure 2 was used: a sealed conical flask as the reaction vessel containing 20 g of MSWFA and 250 ml of 2.5 M NaOH solution and an inverted measuring cylinder submerged in water containing water for the evolved H_2_ gas volume. The displacement of water was measured until there was no further gas generation and using equation (2.2), the Al^0^ wt% (*f_Al_*) in the ash was calculated.

(2.1) $$2Al^{0}\;\left(s\right)+2NaOH\;\left(aq\right)+6H_{2}O\to 2Na\left[Al{\left(OH\right)}_{4}\right]\;\left(aq\right)+3H_{2}\;\left(g\right)$$

(2.2) $$f_{Al}=\frac{\frac{2}{3}\;\times \;\frac{\Delta V}{24}\;\times \;M_{Al}}{m_{ash}}.$$

In equation (2.2), the parameters are: *ΔV* = the volume of hydrogen gas (L); _$M_{A1}$_ = the atomic mass of Al (g mol^−1^); and *m*_ash_ = the mass of the ash sample (g).

#### 2.3.2. Compressive strength

The compressive strength of the granules was detected by selecting ten closely spherical and similarly sized granules from each batch and measuring their diameter (*d* [mm]) with a caliper. The force required to crush them (*F*_max_ [N]) was measured with a Zwick Roell Z010 universal testing machine. Compressive strength *σ* (MPa) was calculated with equation (2.3).


(2.3) $$\sigma =\frac{4\;\times \;F_{max}}{\pi \;\times \;d^{2}}.$$

#### 2.3.3. Bulk density, water absorption and porosity

Bulk density (*ρ*_ssd_ [g cm^−3^]) and water absorption (%) of the granules were determined with a pycnometer. The mass of dried granules (*m*_1_ [g]) was measured, and placed in the pycnometer, which was filled with a known mass of water (*m*_2_ [g]). The pycnometer was kept at constant temperature (22 ± 3°C) for 24 h, then emptied and filled again with a known mass of water (*m*_3_ [g]). The saturated granules were dried at 45°C and weighed (*m*_4_ [g]). The bulk density and water absorption were calculated with equation (2.4) and equation (2.5), respectively. In equation (2.4), *ρ*_w_ [g cm^−3^] is the density of water.


(2.4) $$\rho_{ssd}=\rho_{w}\times \frac{m_{1}}{m_{1}-\left(m_{2}-m_{3}\right)}$$

(2.5) $$Water\;absorption=\;\frac{100\;\times \;\left(m_{1}-m_{4}\right)}{m_{4}}.$$

To observe the porosity of the samples, high-resolution imaging was performed using an X-ray microscope (Zeiss Xradia Versa 610). The scanning parameters were set as follows: a voltage of 60 kV, a power of 10 W and a current of 100 µA, utilizing the Zeiss low energy filter #4 and optical magnification of ×4. The exposure time was set to 7 s, with a total of 2401 projections per 360° rotation. After scanning, the data were reconstructed into 16-bit image stacks with 5.3 µm isotropic voxel size. To reduce noise while preserving edges, an edge-preserving anisotropic filter, available in ImageJ (v. 1.54 f), was applied. The processed image stacks were then converted to an 8-bit format. Porosity analysis was conducted on these stacks using a CTAn software (v. 1.20.3.0, Bruker microCT). Further details on the analysis process are as follows: the samples underwent a binarization process utilizing the automatic Otsu method in three dimensions, creating binary volumes. These volumes were then used to assess porosity, focusing specifically on the regions within the outer edges of the binary volumes.

To support the porosity data, the microstructure of the granules was assessed with an optical microscope (Leica MZ6) equipped with a camera (Leica DFC420).

#### 2.3.4. Specific surface area and pore volume

Specific surface area and pore volume (pores with a diameter <300 nm) were measured using N_2_ gas adsorption–desorption with a Micromeritics ASAP 2020 instrument. The specific surface area was calculated using the Brunauer–Emmett–Teller (BET) isotherm while the Barrett–Joyner–Halenda (BJH) method was used to calculate pore volume from the desorption data.

#### 2.3.5. Leaching

Leaching was evaluated by applying the SFS-EN 12457-2 standard [28]: 10 g of granules with a diameter between 1 and 4 mm were placed in a 250 ml Teflon bottle and 100 g of ultrapure water was added to achieve a liquid to solid ratio of 10. The bottles were mixed in a rotating shaker for 24 h, liquid was separated with a 0.45 µm membrane filter, and the elemental concentrations were detected with an inductively coupled plasma mass spectrometer (XSeries II, Thermo Fisher Scientific) while anions were detected with an ion chromatography system (ICS-2000, Dionex). The leaching of the constituents (*A* [mg kg^−1^]) was calculated with equation (2.6), where *C* is the concentration of a constituent in the eluate (mg L^−1^), *V* is the volume of leachant used (L), MC is the moisture content ratio as a percentage of the dry mass and *m*_D_ is the dry mass of the test portion (kg)


(2.6) $$A=\;\;C\;\times \;\frac{V}{m_{D}}+\frac{MC}{100}.$$

#### 2.3.6. Cation exchange

A comparative experiment to assess the cation exchange property of the granules was conducted with NH_4_Cl solutions (VWR International, Belgium). Before the experiment, granules were rinsed with 0.1 M acetic acid (Merck, Germany) and deionized water to reach neutral pH and dried at 60°C. Another batch of granules was rinsed only with deionized water and dried similarly. Granules (2 g) were placed in 50 m centrifuge tubes, 40 ml of 50 mg L^−1^ NH_4_^+^ solution was added, the tubes were agitated on an orbital shaker with 100 rpm mixing speed at 21°C for 24 h. The solution was separated by filtrating with 0.45 µm membrane filters, it was ensured that pH was in the range of 6−8.5, and the concentration of NH_4_^+^ was measured using an ion-selective electrode (Hach IntelliCAL ISENH4181) and a Hach HQ4100 meter. The cation exchange capacity (*q*_e_ [mg g^−1^]) was calculated with equation (2.7), where *C*_0_ (mg L^−1^) is the initial concentration of NH_4_^+^, *C*_e_ (mg L^−1^) is the concentration of NH_4_^+^ in the solution after 24 h; *V* (L) is the volume of the solution; and *m* (g) is the mass of the granules. Each experiment was performed as a duplicate


(2.7) $$q_{e}=\frac{\left(C_{0}-C_{e}\right)\times V}{m}.$$

## 3. Results and discussion

### 3.1. Compressive strength, density, water absorption and porosity of the granules

**Figure 3.**
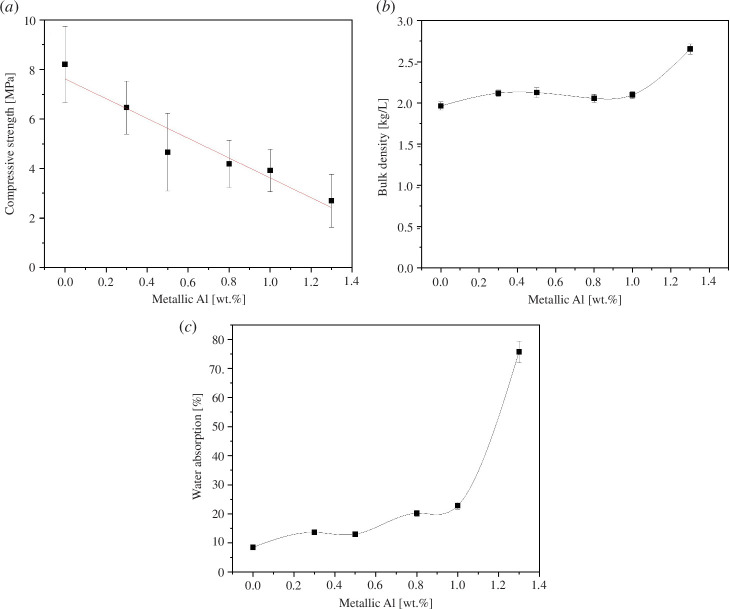
Characterization of granules: (*a*) compressive strength (*n* = 10), (*b*) bulk density (*n* = 5) and (*c*) water absorption (*n* = 5) as a function of Al^0^ introduced by the MSWFA. The error bars represent one standard deviation of the average value.

The compressive strength of the granules decreased linearly as the ash content (and Al^0^ content) introduced by the ash increased (figure 3). Nevertheless, the compressive strength of the granules even with the highest Al^0^ content was in the same range as with light-weight expanded clay aggregates (LECA) with approximately similar diameter [29]. Possible reasons for the decreasing strength upon ash introduction are a lower reactivity of the ash in comparison to metakaolin, lower amount of introduced alkali-activator solution due to the easier granule formation (table 2), and increased porosity upon the hydrogen gas generation from Al^0^ (equation (2.1)). Compressive strength is inversely proportional to the porosity of the material since the pores provide sites for crack initiation and propagation under compressive loading and higher porosity make it easier for stress to concentrate and cause failure. However, bulk density and water absorption (figures 3*b* and *c*) had only minor changes in the range of 0.0−1.0 wt% Al^0^ content while with 1.3 wt% of Al^0^, they increased significantly. Thus, the granules with the highest Al^0^ content were more porous and likely the trend in the compressive strength is more related to the lower reactivity of the ash in comparison to metakaolin or to the introduced amount of the alkali-activator. The higher density of the granules prepared with 1.3 wt% of Al^0^ might be due to the higher density of ash itself compared to metakaolin.

**Figure 4.**
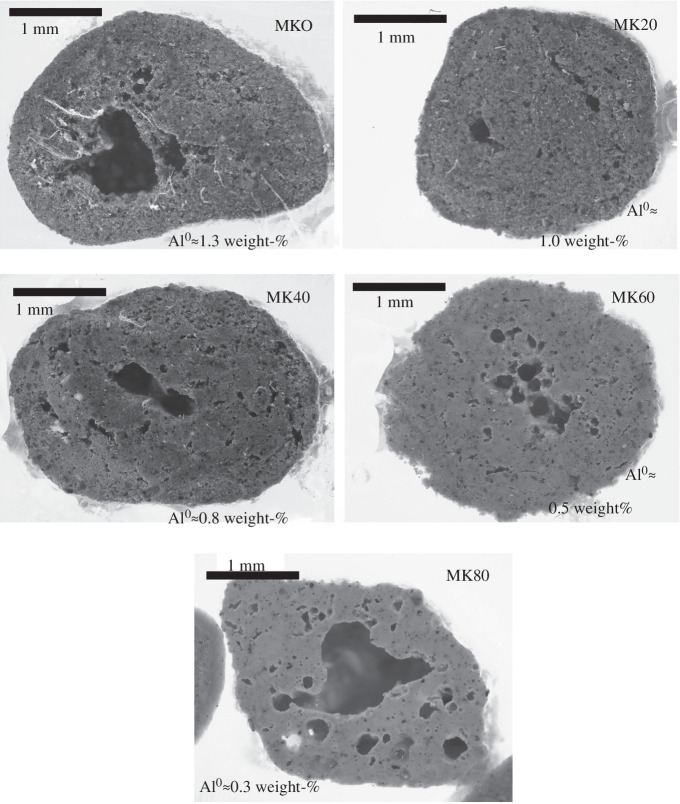
Visual microscopic examination of the cross-sections of the granules.

**Figure 5.**
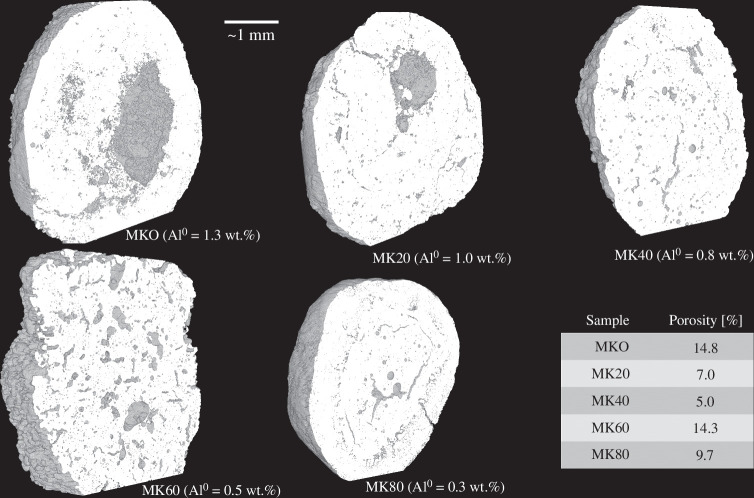
Examples of the granules imaged by X-ray microtomography and quantified porosities: there was no clear correlation between the introduced Al^0^ amount and porosity.

The porosity of the Al^0^-containing granules was visually examined and quantified using light microscopy (figure 4) and X-ray microtomography (figure 5), respectively. As can be seen in figure 4, there were large pores (diameter of approx. 100−1000 µm) located inside the granules while the exterior of the granules was less porous. This indicates that the hydrogen gas bubbles formed upon Al^0^ oxidation were likely merged, forming a hollow interior. Nevertheless, there appears to be no clear correlation with the Al^0^ content and the existence of the large pores: their presence was more evident in MK80 (i.e. with 0.3 wt% of Al^0^) than in MK0 (i.e. with 1.3 wt% of Al^0^). It might be possible that the merging of bubbles is more prone to occur in granules which become more wet in the granulation process which decreases the viscosity of the paste forming the granules. Thus, the formation of the large pores could occur via a random process controlled by the addition of the granulation fluid. The heterogeneity of porosity was also confirmed with the X-ray microtomography (figure 5) where the quantified porosity (detection limit of ~10 µm pore size) varied within 5.0−14.8%. The highest porosities were observed in those granules containing the hollow interior (diameter of mm-scale) or larger pores (diameter of hundreds of µm). Again, there was no clear correlation between the porosity and the introduced Al^0^ amount.

Overall, the analysis of compressive strength, bulk density, water absorption and porosity indicated that already 0.3 wt% of Al^0^ introduction to metakaolin (i.e. 20% of MSWFA) during granulation might be enough from the viewpoint of porosity formation. At that Al^0^ amount, the compressive strength of granules was more than 6 MPa which is sufficiently high for most of the applications.

The specific surface area, micropore volume and total pore volume (considering pores with a diameter of <300 nm) is shown in table 3. The specific surface area and pore volumes have a decreasing trend as MSWFA is introduced to the mixture. The porosity of the samples is in the meso- or macropore region as can be seen from the very low micropore volume.

**Table 3.** Specific surface area, micropore volume and total pore volume of the granules.

sample ID	specific surface area [m^2^ g^−1^]	micropore volume [cm^3^ g^−1^]	total pore volume (for pores with a diameter < 300 nm) [cm^3^ g^−1^]
MK100	27.9	0.001	0.104
MK80	18.6	0.001	0.133
MK60	20.0	0.001	0.126
MK40	17.5	0.000	0.148
MK20	11.0	0.000	0.084
MK0	5.2	0.000	0.048

### 3.2. Leaching

To further evaluate the application possibilities of the granules (samples MK20–MK100), a leaching experiment was conducted to assess the release of metal(loid)s and anions. The leaching results were compared to the Finnish legislative limits of earth construction applications (table 4). All other metal(loid)s and anions were within the limits for all samples except antimony (Sb), vanadium (V), chloride (Cl^−^) and sulphate (SO_4_^2−^). However, the leached amount of Sb, Cl^−^ and SO_4_^2−^ decreased upon increasing the MK content of the granules and for the MK80 sample, only Sb and V remained higher than the limit. For vanadium, the trend was reversed, and the increase in the MK content caused higher leaching of vanadium. Similar results have been reported earlier, that is, the leaching of vanadium can be relatively high from MK-based geopolymers [30]. Furthermore, it has been also reported that the leaching of oxyanions or negatively charged hydroxide species (e.g. As or Sn) may increase when MK was introduced to a mixture of MSWFA and cement [31]. Thus, the leaching results are in line with the existing literature, but they suggest that the alkali-activated MK-MSWFA granules are not suitable for direct use in earth construction. Rather, they could be considered as artificial light-weight aggregates for concrete where the surrounding binder phase would provide additional stabilization. However, it should also be noted that vanadium is a trace component in MK and its leaching will most likely decrease quickly after the initial exposure to water. Similarly, the leaching of Sb from the MK80 granules also likely represents a high initial value which will decrease upon being flushed with water.

**Table 4.** Leaching of metal(loid)s and anions from granules with a comparison to the legislative limits of earth construction in Finland (i.e. a coated road with a max. thickness of the coating material 1.5 m) according to the Finnish Government decree 843/2017. The values exceeding the limits are italicized.

parameter	MK20	MK40	MK60	MK80	MK100	legislative limit
pH	11.34	11.57	11.72	11.74	11.38	—
conductivity [mS cm^−1^]	15.15	12.19	9.351	6.325	2.734	—
Al [mg kg^−1^]	13	32	170	280	420	—
As [mg kg^−1^]	0.23	0.48	0.98	1.2	0.28	2
Ba [mg kg^−1^]	0.54	0.21	<0.06	<0.06	<0.06	100
Cd [mg kg^−1^]	<0.002	<0.002	<0.002	<0.002	<0.002	0.06
Co [mg kg^−1^]	<0.004	<0.004	<0.004	<0.004	<0.004	—
Cr [mg kg^−1^]	6.3	7.1	4.3	4.1	0.38	10
Cu [mg kg^−1^]	0.013	<0.01	0.012	0.014	0.019	10
Fe [mg kg^−1^]	<0.5	<0.5	<0.5	0.62	0.83	—
Mo [mg kg^−1^]	4.6	4.5	2.9	2.2	0.19	6
Ni [mg kg^−1^]	0.019	<0.01	<0.01	<0.01	0.015	2
Pb [mg kg^−1^]	0.0059	0.007	0.0099	0.0087	0.0042	2
Sb [mg kg^−1^]	*2.6*	*2.8*	*2.1*	*1.7*	<0.01	0.7
Se [mg kg^−1^]	0.28	0.44	0.34	0.25	<0.04	1
Ti [mg kg^−1^]	<0.15	<0.15	<0.15	<0.15	<0.15	—
V [mg kg^−1^]	*3.5*	*7.3*	*10*	*22*	*24*	3
Zn [mg kg^−1^]	0.27	0.13	0.23	0.27	0.24	15
Hg [mg kg^−1^]	<0.004	<0.004	<0.004	<0.004	<0.004	0.03
dissolved organic carbon [mg kg^−1^]	63	35	46	44	57	500
F^−^ [mg kg^−1^]	44	56	70	52	20	150
Cl^−^ [mg kg^−1^]	*26 000*	*19 000*	11 000	5300	63	11 000
SO_4_^2−^ [mg kg^−1^]	*41 000*	*30 000*	*19 000*	9900	50	18 000
total dissolved solids [mg kg^−1^]	1 00 000	78 000	56 000	36 000	9600	—

### 3.3. Cation exchange capacity

AAMs are actively studied as adsorbents [32], and thus it was evaluated whether the alkali-activated MK-MSWFA could have suitable adsorption properties as a cation exchanger. The porosity introduced by Al^0^ could be beneficial in the application as adsorbents [22]. It should be noted that the cation exchange experiment does not provide the maximum cation exchange capacity but a comparative NH_4_^+^ uptake under the experimental conditions (i.e. pH of ~7, granule dosing 2 g/40 ml, initial NH_4_^+^ concentration of 50 mg L^−1^ and mixing time of 24 h at 21°C).

**Figure 6.**
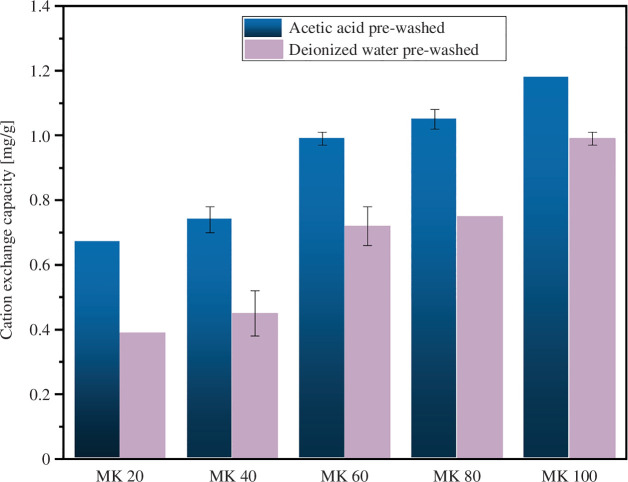
Comparative cation exchange amount of the granules under the experimental conditions (pH of ~7, granule dosing 2 g per 40 ml, initial NH_4_^+^ concentration of 50 mg L^−1^ and mixing time of 24 h at 21°C). The results are shown as an average (*n* = 2) and the error bars represent the difference between the measurements.

MK100 granules pre-washed with acetic acid adsorbed 1.18 mg g^−1^ of NH_4_^+^ and had the best performance in the NH_4_^+^ removal. However, as the MSWFA content of the granules was increased (i.e. samples MK80–MK20), the NH_4_^+^ uptake decreased (figure 6). Nevertheless, MK80 still had a satisfactory performance as a cation exchanger (~1.05 mg g^−1^ NH_4_^+^ uptake). A possible explanation for the decrease in the adsorption amount is the introduction of calcium from the MSWFA to the granules: it has been earlier shown that high-Ca AAMs have a lower NH_4_^+^ uptake compared to the low-Ca AAMs [33]. The NH_4_^+^ adsorption is considered to occur on the tetrahedral Al sites of the geopolymer or AAM structure [34] and MSWFA has a much lower Al content than MK (table 1) which decreases the NH_4_^+^ uptake. Another contributing factor may be the decreasing specific surface area and pore volume (of pores <300 nm) upon introducing MSWFA to the granules as shown in table 3. The maximum cation exchange capacity of finely powdered MK-based geopolymers has been reported to be 32−87 mg g^−1^, depending on the MK composition and sample age [7,33]. Finally, it should be noticed that the acetic acid pre-washed granules resulted in a higher NH_4_^+^ cation exchange amount in comparison to granules washed with only deionized water. The higher adsorption capacity of acetic acid-washed samples could be due to the more efficient removal of unreacted alkali activator, improved meso- and micro-porosity and replacement of Na^+^ by H^+^ on the tetrahedral Al sites [35]. On the other hand, using mild acetic acid (0.1 M) does not result in dealumination as much lower pH would be required for that [22,36].

## 4. Conclusions

In this study, MSWFA was combined with metakaolin at different ratios ranging from 0% to 100% as MSWFA and granulated in a high-shear granulator using aqueous sodium silicate as the granulation fluid. The obtained granules were porous due to the metallic Al introduced by the MSWFA. The granules were characterized for their morphology, porosity, mechanical strength, leaching and cation exchange capacity. As the MSWFA content increased, there was an increase of metallic Al and the alkali-activated phase turned from metakaolin geopolymer to alkali-activated MSWFA. In terms of compressive strength, all granules (including 100% MSWFA) had sufficiently high value (i.e. ≥2 MPa) for most of the practical applications. Porosity of the granules (indicated by water absorption) increased as the MSWFA content increased but there was a leap when increasing MSWFA from 80% to 100%. However, when the porosity was determined with light microscopy or X-ray microtomography, there was no clear correlation between the MSWFA content and observed porosity. However, this does not exclude presence of much finer pores. In terms of the porosity formation, already 0.3 wt% of Al^0^ (i.e. 20% of MSWFA) was enough. The developed granules had higher leaching of antimony (Sb), vanadium (V), chloride (Cl^−^) and sulphate (SO_4_^2−^) than allowed by the Finnish legislation for materials intended to earth construction. The leaching of Sb, Cl^−^ and SO_4_^2−^ diminished upon increasing the metakaolin content and when using 20 wt% of MSWFA (i.e. 0.3 wt% of Al^0^), only Sb and V remained over the limit. However, for V, the trend was opposite, and the source of V was identified as the metakaolin used in the experiments. It should be noted though that the leaching experiment indicated only the initial leaching which likely decreases over continued exposure to water. Finally, the cation exchange experiment indicated a decreasing performance upon increasing the MSWFA content, likely due to the decrease of available tetrahedral Al sites, specific surface area and pore volume. However, the ash content of 20% (i.e. 0.3 wt% of Al^0^) still had an acceptable cation exchange performance. As an overall conclusion, the granules developed in this study might be best suited as light-weight artificial aggregate used in concrete: there, the additional binder would provide additional stabilization for the leaching issues.

## Data Availability

The raw data required to reproduce these findings are available for download from [37].
